# 

**Published:** 1912-06-15

**Authors:** 


### THE DENTAL REGISTER

Edited by Dr NELVILLE S. HOFF
ANN ARBOR, MICH.

VOL. LXVI. JUNE, 1912 NO. 6.

THE SAMUEL A. CROCKER CO., Publishers
OHIO DENTAL AND SURGICAL DEPOT
35 W. FIFTH STREET, CINCINNATI, O.

PRICE, ONE DOLLAR A YEAR

Entered at the Post-Office at Cincinnati, O., as second-class mail matter

